# Prospective cross-sectional study on the knowledge and practice of patients visiting outpatient urology clinic in Nigeria on coronavirus disease

**DOI:** 10.11604/pamj.2022.43.40.31864

**Published:** 2022-09-26

**Authors:** Ehiremhen Ozah, Eshiobo Irekpita, Ugochukwu Alili, Vincent Chukwudi Onuora

**Affiliations:** 1 Urology Unit, Department of Surgery, Irrua Specialist Teaching Hospital, Irrua, Edo State, Nigeria,; 2 Department of Surgery Urology Division Ambrose Alli University, Ekpoma Edo State, Nigeria

**Keywords:** COVID-19, disease, health care, hospital, knowledge, practice, urology, outpatient, ISTH

## Abstract

**Introduction:**

coronavirus disease is caused by a respiratory virus (severe acute respiratory syndrome coronavirus 2). It has assumed a health burden worldwide; hence it was declared a public health emergency of international concern. The pandemic has affected all health related disciplines; urology practice is not spared. It has also had its toll on socioeconomic life. The study aims to assess the knowledge and practice of patients visiting outpatient urology clinic on coronavirus disease (COVID-19).

**Methods:**

this is a cross-sectional study conducted to assess the knowledge and practice of patients visiting outpatient urology clinic on COVID-19. All patients aged 18 years and above who consented were included. Association between independent variables (social demographic characteristics) and dependent variables (knowledge and practice) using Fisher´s exact, while predictors of dependent variables were assessed using logistics regression model.

**Results:**

a total of 154 respondents participated. Majority of respondents (50.0%) were between 60 and 69 years. Male constitute 90.3% of respondent. Majority, 65% of respondent had good knowledge while (80%) adopted good practice towards preventing COVID-19. There was an association between age and practice of preventive measure (p = 0.032). There were no predictors of knowledge and practice of preventive measures towards coronavirus disease amongst the socio-demographic characteristics.

**Conclusion:**

concerted efforts should be made at educating the populace on knowledge, good practices across socio-demographic groups on COVID-19, particularly at the Hospital. Measures should be in place to develop Tele-health as this would improve response at curtailing coronavirus disease and improve health care.

## Introduction

Coronavirus infections are evolving. They are respiratory viruses known to cause illness ranging from common cold to severe acute respiratory syndrome (SARS) [1]. Coronavirus are zoonotic pathogens that can be transmitted from animals to human and from human to human [2]. Epidemic outbreaks resulting from coronaviruses have been witnessed in the past, beginning from 2002 (SARS) with 800 deaths [2]. Ten years after the Middle East respiratory coronavirus (MERS-CoV) with 860 deaths occurred, with the first case of MERS occurring in Jordan in April 2012. Eight years after MERS-CoV epidemic, the world is standing still for yet another outbreak, the coronavirus disease, COVID-19 [3]. It was reported as pneumonia of unknown origin to the WHO country office in China on 31^st^ December 2019 [4]. The current outbreak of novel coronavirus (COVID-19) in Wuhan city, Hubei province of China was declared by World Health Organisation (WHO) as a public health emergency of international concern (PHEIC) on 30^th^ January 2020 [5]. As of 30^th^ April 2020, globally there had been 3,090,445 confirmed cases across 214 countries and territories with 217,769 fatalities, with Nigeria witnessing 1,532 confirmed cases and 44 deaths during the period.

Mode of spread identified include human to human transmission through droplets, feco-oral and direct contact, with an incubation period of 2-14 days [6]. It is prudent during this pandemic in the absence of extensive community testing and effective isolation and quarantine strategies in place that health professionals perform their duty with the presumption that all patients they treat are potentially infected with COVID-19, even if asymptomatic, given that high infection rates in asymptomatic individuals have been documented [7]. Elderly patients and those with underlying conditions are at greater risk of severe infection and fatality due to COVID-19 [8]. In this regard, it is necessary not to consider only the risk to staff but patients as well, thus reducing unnecessary frequent outpatient visit is pertinent. Since there is clinical evidence that SARS-CoV-2 is present in stools of COVID-19 patients, transmission during various procedures (i.e. transrectal prostate biopsy, urinary diversion) might be possible [9]. Duration of virus shedding of COVID-19 virus in urine is unknown [10]. A recent study by Ling *et al*. reported limited persistence of SARS-CoV-2 nucleic acid in urine [11]. Even though not yet documented, transmission during urine sampling (e.g. for urine m\c\s) has been found possible because of which urethral catheterization should be carried out with caution. Emphasis should be on preventive measure, which is critical to limiting spread of COVID-19 infection, while the world await development of a vaccine and validated potent antiviral therapy. World Health Organisation (WHO) and centre for disease control and prevention (CDC) have made recommendations for prevention and control of COVID-19 for healthcare workers and the general population [12,13].

In a bid to curtail transmission of COVID-19, health literacy is an important tool. It is a key determinant of health and accounts for health disparities by age, race/ethnicity and socioeconomic status [14]. Health literacy profile has proved that better sociodemographic status positively influenced health literacy [15-18]. In terms of age, researchers have reported that health literacy changes dynamically with age [19]. A Statistically significant association had been found between health literacy level and health related behavior including timing of seeking medical services, respondents aged 60-74 years had a higher health literacy than those above 75 years (Odds ratio= 2.06, 95% CI: 1.23-3.42; P= 0.006) in a recent study carried out in China [20]. The study also found out that the literacy level of respondents living in urban areas was higher than those living in rural areas with an odds' ratio of 3.28; p<0.001, it was also noted that the probability of having better literacy level in group who have completed secondary education than those who did not (odds ratio= 2.86, 95% CI: 1.92-4.27, P<0.001) [20]. Health information could influence individuals to take health promoting action [21,22]. The outpatient urology clinic of Irrua Specialist Teaching Hospital (ISTH) in the past attends to an average of 60 patients per clinic day. The advent of coronavirus disease has seen a decline in patient traffic. The hospital management and urology unit have put in place far-reaching measures to prevent spread of COVID-19 infections like screening of healthcare workers and patients using infrared thermometers, handwashing, use of alcohol based hand rub, social distancing, reduction in consultation time per patient, triaging of patients to determining duration of clinic appointments. This work aims to assess the knowledge, practice and impact of coronavirus disease (COVID-19) on patients visiting the outpatient urology clinic at Irrua Specialist Teaching Hospital (ISTH).

## Methods

**Study design, setting and participants:** this is a cross-sectional descriptive study conducted using a self-administered structured questionnaire. The study was carried out at Irrua specialist Teaching Hospital, Irrua, Edo State, a 400 bed capacity hospital established as tertiary health care delivery center, offering a range of specialist urology services. It is also a designated infectious disease referral Centre. All patients 18 years and above who consented to participate within the study period presenting in an outpatient urology clinic were selected for the study. The only exclusion criteria were failure to give consent and the age limit of 18 years. The study was carried out between June and November 2020.

**Sample size determination:** the sample size was calculated using the formula:


$$n=\frac{z^{2}pq}{d^{2}}(D)$$

Where n = desired sample size, z= level of significance at 95% CI (1.96), p is cumulative incidence of COVID-19 at 0.056 [23], q= 1-p = 0.944 and d= degree of accuracy desired, usually set at 0.05, while D is design effect put at 1.5. The minimum sample size required for the study was 122.

**Data collection:** a pretested questionnaire developed by the authors as a new tool consists of 4 sections. In the first section, participants were informed about the objective of the study. They were also informed that despite consent to participate, they can decide to withdraw from the study if they deem it necessary. Participants were also informed that information and opinions giving would be anonymous and confidential. Written informed consent was also obtained in the first section of the questionnaire. The second section consist of demographic variables (age, sex, religion, ethnicity, marital status, occupation and level of education), the third section had variables on knowledge encompassing 19 items on knowledge on COVID-19, which include awareness of COVID-19, source of information, where it was first diagnosed, symptoms, asymptomatic carriers, preventive measures, information on therapy, isolation and its effectiveness, while the fourth section had 8 variables, items were on practice towards COVID-19 on urologic care which include measures adopted towards prevention. Data collected by using a structured questionnaire which was self-administered by patients visiting an outpatient, urology clinic who gave consent and met the inclusion criteria. To ensure confidentiality, the names of participants and case note numbers were excluded from the questionnaire.

**Validity of questionnaire:** this questionnaire was developed based on CDC guidelines [12,13], 19 items on knowledge and 5 items on practice, participant were expected to respond to knowledge and practice questions either by True or False and an additional “I don´t know” option. A score of one was given for an appropriate response and a score of zero for an inappropriate answer, or I don´t know answer. A high score indicated good knowledge and good practice based on the composite score for knowledge and practice respectively. Items were evaluated for internal reliability, using Cronbach´s alpha [24]. Cronbach´s alpha coefficient was 0.70, indicating internal reliability. The tool had not been previously validated.

**Data analysis:** statistical analysis was done IBM SPSS (statistical package for the social sciences) software version 21. Numerical data, normally distributed such as age (years) were expressed in mean ± standard deviation. Categorical data such as (sex, marital status, level of education, religion, ethnic group, dependent variables) were expressed as frequencies and percentages. Association between independent variables (social demographic characteristics) and dependent variables (knowledge and practice) were assessed using Fisher´s exact in a bivariate analysis; while predictor of dependent variables were assessed using logistic regression model in a multivariate analysis this was done to avoid confounding effects by analyzing all variables together, steps were taking to ensure that the dependent variables were categorized and that the outcome (dependent) variable were dichotomous. The level of significance was set at p<0.05.

**Ethical approval:** all eligible participants were informed about the aims of the study; consent was signed before participation. The protocols of the study were approved by the Hospital research and ethics committee (HREC) of Irrua specialist Teaching Hospital. The protocol number for the work is ISTH/HREC/20200506/074. Participants were informed of their right to withdraw from study at any time they wish without any consequence.

## Results

The study was carried out between June and November 2020. Following ethical approval and consent from the participants, 154 consecutive patients presenting in the outpatient urology clinic within this period who gave consent participated. The age range of respondents was between 18-87 years. Seventy-seven (50.0%) respondents were between the ages of 60 - 69 years, while 28 (18.2%) were between the ages of 70-79 years. The mean (SD) age is 61.9 (12.9). One hundred and thirty-nine (90.3%) respondents were males, while 15 (9.7%) were females. One hundred and thirty (84.4%) respondents were married, while 13 (8.4%) respondents were single. Eighty-eight (57.1%) respondents had tertiary education, while 31 (30.1%) had primary education. Using the international classification of occupation, 34 (22.1%) respondents had occupational skill level 2 while 27 (17.5%) respondents had occupational skill level 3. One hundred and twenty-nine (83.8%) respondents were Christian, while 20 (13.0%) respondents were Muslim. Forty-seven (30.5%) respondents were Esan while 11 (30.5%) were Bini as shown in Table 1. One hundred and fifty (97.4%) respondents have heard of COVID-19. One hundred and thirty-six (88.3%) respondents believed COVID-19 was first diagnosed in China while 143 (92.9%) agreed it can be transmitted. Sixty-five (50%) choose droplet as a means of transmission, while 36 (27.7%) chose direct contact. A hundred and twenty (77.9%) chose fever as a symptom, 115 (74.7%) chose cough and sneezing as a symptom of COVID-19, while 79 (51.3%) chose shortness of breath. Ninety-seven (63.0%) agreed it can be transmitted by asymptomatic carrier. One hundred and seven (69.5%) agreed it is hospital acquired, 126 (81.8%) respondents were not aware of any therapy. One hundred and twenty-nine (83.8%) respondents were aware it is of public health concern, 134 (87.0%) respondents agreed isolation is an effective way to reduce spread while 135 (87.7%) respondents agreed people in contact with COVID-19 patients should be quarantined for 14 days. Majority of participants (65%) demonstrated good knowledge of COVID-19. There was no determinant of knowledge of COVID-19 among the socio-demographic characteristics of the respondents, age of respondent and knowledge using Fisher´s exact showed p=0.280 which was not statistically significant, while the association between sex and knowledge showed a p value of 0.395 using Chi-square, which was also not significant statistically. Marital status, level of education, religion and ethnic group had p- values of 0.310, 0.089, 0.641 and 0.315 respectively which were not significant statistically, using Fisher´s exact, while occupational skill level had a p-value that was statistically insignificant using Chi-square p=0.319 as shown in Table 2.

**Table 1:** socio-demographic characteristics of respondent

Variable	Frequency (n =154)	Percent
**Age (years)**		
<20	1	0.6
20 - 29	4	2.6
30 - 39	6	3.9
40 - 49	7	4.5
50 - 59	23	14.9
60 - 69	77	50
70 - 79	28	18.2
80+	8	5.2
**Sex**		
Male	139	90.3
Female	15	9.7
**Marital status**		
Single	13	8.4
Married	130	84.4
Widowed	11	7.2
**Level of education**		
No formal education	5	3.2
Primary	30	19.5
Secondary	31	20.1
Tertiary	88	57.1
**Occupational level**		
Skill level 1	23	14.9
Skill level 2	34	22.1
Skill level 3	27	17.5
Skill level 4	16	10.4
*No information	54	35.1
**Religion**		
Christian	129	83.8
Muslim	20	13.0
African traditional religion	3	1.9
Atheist	2	1.3
**Ethnic group**		
Esan	47	30.5
Bini	11	7.1
Etsako	9	5.8
Delta	6	3.9
**Others	33	21.4
*No information	48	31.2

Mean ± SD age = 61.9 ± 12.9 years

**Table 2:** association between socio-demographic characteristics and knowledge

Variable	Frequency n (%)		Test statistics	P-value
	Poor	Good		
**Age (years)**				
<20	0 (0.0)	1 (1.0)	8.278**††**	0.280
20 - 29	1 (1.9)	3 (3.0)		
30 - 39	1 (1.9)	5 (5.0)		
40 - 49	3 (5.6)	4 (4.0)		
50 - 59	4 (7.4)	19 (19.0)		
60 - 69	28 (51.9)	49 (49.0)		
70 - 79	12 (22.2)	16 (16.0)		
80+	5 (9.3)	3 (3.0)		
**Sex**				
Male	47 (87.0)	92 (92.0)	0.982**†**	0.395
Female	7 (13.0)	8 (8.0)		
**Marital status**				
Single	2 (3.7)	11 (11.0)	2.348**††**	0.310
Married	48 (88.9)	82 (82.0)		
Widowed	4 (7.4)	7 (7.0)		
**Level of education**				
No formal education	4 (7.4)	1 (1.0)	6.177**††**	0.089
Primary	13 (24.1)	17 (17.0)		
Secondary	11 (20.4)	20 (20.0)		
Tertiary	26 (48.1)	62 (62.0)		
**Occupational level**				
Skill level 1	11 (28.9)	12 (19.4)	3.546**†**	0.319
Skill level 2	13 (34.2)	21 (33.9)		
Skill level 3	11 (28.9)	16 (25.8)		
Skill level 4	3 (7.9)	13 (21.0)		
**Religion**				
Christian	47 (87.0)	82 (82.0)	1.960**††**	0.641
Muslim	6 (11.1)	14 (14.0)		
African traditional religion	0 (0.0)	3 (3.0)		
Atheist	1 (1.9)	1 (1.0)		
**Ethnic group**				
Esan	17 (47.2)	30 (42.9)	4.728**††**	0.315
Bini	1 (2.8)	10 (14.3)		
Etsako	4 (11.1)	5 (7.1)		
Delta	1 (2.8)	5 (7.1)		
Others	13 (36.1)	20 (28.6)		

† †Fishers Exact;†Chi-Square

**Figure 1:**
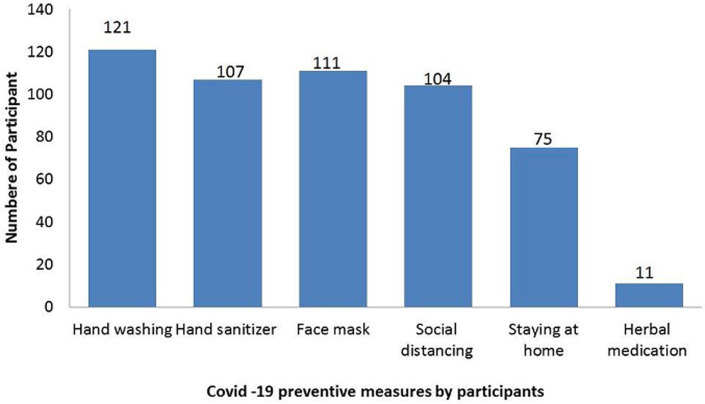
preventive measure

One hundred and twenty-one respondents chose handwashing as the preventive measure they observe, 107 respondents chose application of hand sanitizer, 111 respondents chose face mask, 104 respondents chose social distance, 75 respondents chose to stay at home and 11 respondents chose herbal medication (Figure 1). Table 3 shows that the odds' ratio for age (0.977; p-value 0.134) as a predictor of knowledge of COVID-19 was statistically not significant, while the odds' ratio for sex (0.627; p-value 0.414) as a predictor of knowledge of COVID-19 was statistically not significant. Marital status as predictor of knowledge of COVID-19 revealed that participants who were married had an odds' ratio (0.311; p-value 0.139), widowed had an odds' ratio (0.318; p-value 0.248) which was statistically not significant, the odds' ratio for level of education and occupational skill level as predictor of knowledge of COVID-19 was also not statistically significant. One hundred and twenty (77.9%) respondents always wash their hands while 20 (13.0%) respondents wash their hands more than half of the time, 119 (77.3%) respondents have bought hand sanitizer and 103 (66.9%) respondents always use hand sanitizer while 26 (16.9%) respondents use hand sanitizer more than half of the time. One hundred and eight (70.1%) respondents have been attending their follow-up clinic. Majority (86%) respondents had good practice of preventive measures about COVID-19. Age of respondent (P-value = 0.032) and ethnic group (P-value = 0.012) were the only determinant of practice of preventive measure of COVID-19 (Table 4). As shown in Table 5, the odds' ratio of age (1.033; p-value 0.052) as a predictor of practice of safety measures to prevent COVID-19 was not statistically significant, while the odds' ratio for sex (1.035; p-value 0.966) was also not statistically significant. Odds ratio for educational level and occupational skill level as predictor of practice of safety measures to preventing COVID-19 was not statistically significant among participants.

**Table 3:** predictors of knowledge of COVID-19

Variables	B (univariate regression coefficient) Unadjusted odd ratio	P-value	Multivariate regression( adjusted-Odds ratio)	95% C.I. for Odds ratio	
				Lower	Upper
Age of respondents	-0.023	0.134	0.977	0.948	1.007
Sex					
Male			1		
Female	-0.467	0.414	0.627	0.204	1.922
Marital status					
Single			1		
Married	-1.169	0.139	0.311	0.066	1.461
Widowed	-1.145	0.248	0.318	0.046	2.223
Level of education					
No informal			1		
Primary	1.477	0.213	4.381	0.428	44.890
Secondary	1.671	0.165	5.316	0.504	56.095
Tertiary	1.881	0.107	6.562	0.667	64.569
Occupation					
Skill level 1			1		
Skill level 2	0.393	0.473	1.481	0.507	4.323
Skill level 3	0.288	0.615	1.333	0.434	4.094
Skill level 4	1.379	0.071	3.972	0.888	17.775

**Table 4:** association between socio-demographic and practice of safety measures

Variable	Frequency n (%)		Test statistics	P-value
	Poor	Good		
**Age of respondent**				
<20	1 (4.5)	0 (0.0)	13.508††	0.032
20 - 29	3 (13.6)	1 (0.8)		
30 - 39	1 (4.5)	5 (3.8)		
40 - 49	0 (0.0)	7 (5.3)		
50 - 59	2 (9.1)	21 (15.9)		
60 - 69	10(45.5)	67 (50.8)		
70 - 79	4 (18.2)	24 (18.2)		
80+	1 (4.5)	7 (5.3)		
**Sex**				
Male	20 (90.9)	119 (90.2)	0.012†	1.000
Female	2 (9.1)	13 (9.8)		
**Marital status**				
Single	4 (18.2)	9 (6.8)	4.147††	0.098
Married	18 (81.8)	112 (84.8)		
Widowed	0 (0.0)	11 (8.3)		
**Level of education**				
No formal education	0 (0.0)	5 (3.8)	1.067††	0.835
Primary	4 (18.2)	26 (19.7)		
Secondary	6 (27.3)	25 (18.9)		
Tertiary	12 (54.5)	76 (57.6)		
**Occupational level**				
Skill level 1	3 (30.0)	20 (22.2)	1.395††	0.759
Skill level 2	2 (20.0)	32 (35.6)		
Skill level 3	3 (30.0)	24 (26.7)		
Skill level 4	2 (20.0)	14 (15.6)		
Religion				
Christian	18 (81.8)	111 (84.1)	2.571††	0.417
Muslim	3 (13.6)	17 (12.9)		
African traditional religion	0 (0.0)	3 (2.3)		
Atheist	1 (4.5)	1 (0.8)		
**Ethnic group**				
Esan	4 (23.5)	43 (48.3)	11.248††	0.012
Bini	6 (35.6)	5 (5.6)		
Etsako	1 (5.9)	8 (9.0)		
Delta	1 (5.9)	5 (5.6)		
Others	5 (29.4)	28 (31.5)		

† †Fishers Exact; †Chi-square

**Table 5:** predictors of practice of safety measures

Variables	B(Univariate regression coefficient) Unadjusted odd ratio	P-value	Multivariate regression (adjusted-Odds ratio)	95% C.I. for Odds ratio	
				Lower	Upper
Age of respondents	0.032	0.052	1.033	1.000	1.067
**Sex**					
Male			1		
Female	0.034	0.966	1.035	0.215	4.972
**Marital status**					
Single			1		
Married	1.074	0.101	2.928	0.811	10.569
Widowed	1.587	0.188	4.889	0.416	51.869
**Level of education**					
No informal			1		
Primary	0.550	0.661	1.733	0.148	20.232
Secondary	-0.182	0.878	0.833	0.082	8.519
Tertiary	0.236	0.836	1.267	0.136	11.800
**Occupation**					
Skill level 1			1		
Skill level 2	0.875	0.360	2.400	0.368	15.640
Skill level 3	0.182	0.834	1.200	0.218	6.613
Skill level 4	0.049	0.960	1.050	0.155	7.127

**Figure 2:**
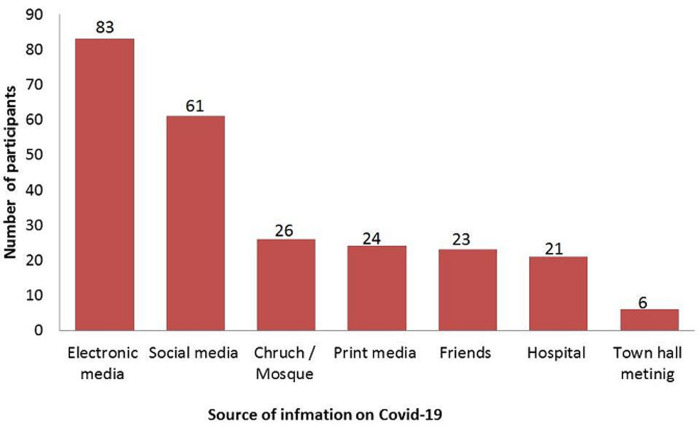
source of information

One-hundred and thirty-five (87.7%) of the respondent will present in the hospital if there are symptoms, 80 (51.9%) respondent will accept self-isolation, while 64 (41.6%) respondents will accept isolation center. One-hundred and twenty (77.9%) respondents were not aware of any patient who has been cleared. One-hundred and ten (71.4%) respondent will not keep an appointment if there is one. Ninety-five (61.7%) respondents will most likely disobey the staying at home restriction, while 37 (24.0%) respondents will most likely disobey the social distance restriction. Seventy-four (48.1%) respondent suggested phone calls as a way of improving out-patient care during the pandemic, while 25 (16.2%) respondents suggested home visit as a way of improving out-patient care during the pandemic. Considerable information from participants on COVID-19 was from the electronic media in 83(34.01%) participants, while social media served as source of information in 61 (25%) of the participants. Hospital and town hall meetings contributed the least to information respondents have on COVID-19 in 8.6% and 2.45% of cases respectively (Figure 2).

## Discussion

The advent of COVID-19 with its attendant impact on health resources and the socio-economic sphere is immense. These impact, and the consequent morbidity and mortality arising from the pandemic, has made it necessary to adopt effective measure for disease prevention. This is imperative considering the fact that there is no effective drug treatment and that vaccines are being developed. The study sought to assess knowledge of patients visiting outpatient´s urology clinic on COVID-19, including practices adopted by these patients seeking urologic care. The study is designed to add value to care by ensuring patients are equipped with appropriate knowledge, which are translated to healthy practices with positive impact on health care in the face of the pandemic. This cross-sectional study demonstrated that a significant percentage of respondents (65%) had good knowledge of COVID-19; this finding was consistent with similar study [25], amongst chronic illness patients in Vietnam. Some research work on knowledge, attitude and practice on COVID-19 has been published in the literature, these are online based survey, most of the respondents in these studies (90%) demonstrated adequate knowledge on COVID-19 [26-29]. The disparity in knowledge in this study and the online based study could be attributed to the fact that these researches were online based and most respondents were knowledgeable to access information on the internet [26-28]. There was no association between socio demographic characteristics of respondents as determinant of knowledge, as revealed in this study using a Fisher´s exact. Logistic regression model also depicted that socio demographic characteristics of respondents were not predictors of knowledge on COVID-19. This finding maybe as a result of availability of information about COVID-19 across socio demographic groups, the majority of respondent (34.01%) got information from electronic media, while social media accounted for 25% of information and hospital education accounted for 8.6% of information. This study is at variance with online based studies where most of the information on COVID-19 came from social media [26-29].

In this study, knowledge was limited on the mode of transmission as only 50% and 16.9% were aware that it could be spread by droplets and that it is airborne respectively. Respondents were also not abreast with knowledge of symptoms associated with COVID-19 such as Anosmia and loss of taste (7.1%) diarrhea (5.8%), headaches, sore throat and malaise, 29.2%, 36.5% and 5.8% respectively. Similar findings were documented in studies [30,31] carried out in South West Nigeria and Iran on mode of transmission and symptomatology. A significant percentage of respondents (86%) in this study adopted practical preventive measures which included handwashing, use of hand sanitizers, face mask, social distancing and avoiding public gatherings. This is a reflection of positive attitudinal and behavioral modifications developed to break the chain of transmission and flatten the COVID-19 curve. These were all translated to good practices in the fight against COVID-19 pandemic. These findings were consistent with what was documented in several other studies [26,28-30] perhaps; knowledge is a pre-requisite for promoting preventive measures and forming positive attitude towards the fight against COVID-19 [32].

Furthermore, age was found to be a determinant to practice of preventive measures in this study (p-value=0.032) which was statistically significant using Fisher´s exact. The likely reason may be due to the fact that ageing is considered a risk factor for severity of coronavirus disease and mortality is more in the ageing population [8]. However, logistic regression model revealed that there was no predictor to the practice of preventive measures of COVID-19 amongst the socio demographic characteristics of the respondents. However, despite the adoption of good practices by respondent, the majority of respondent (61.7%) affirmed that they are likely to disobey stay at home order (avoiding social gathering), this agrees with observation made in previous study [26] in North Central Nigeria. Amongst respondents in this study, most (87.5%) would present in the hospital, if they have symptoms suggestive of COVID-19, only 41.6% will accept to be treated in isolation, these may have been a measure to prevent stigma. A considerable proportion of respondent (71.4%) also will not keep appointment with anyone who has been managed for COVID-19, transmitting to discriminating attitude towards COVID-19 survivors, this finding contrast revelation in the study conducted in South West Nigeria [30] Stigma generates negative impact on its victims unchecked stigma can lead to dire psychosocial comorbidities, the risk of psychiatric disorders and suicidal tendencies being one of them [33].

**Strengths:** study is unique because patients rather than health workers formed the basis upon which it was contrived, the outcome would further strengthen health education policies. It also derived its strength from being a prospective study, hence elements of bias were limited.

**Limitations:** the statistical power of the study was limited by the study population, since hospitals across the world witnessed a major decline in patients´ traffic during the period of the study. A community based study that will add more statistical power to generalizations will be carried out in future. This will also assess how telemedicine can improve practice and preventive measures. The construct of knowledge and practice were adapted to urologic patient population hence the outcome cannot be generalizable to the general population, while the scale used to measure overall knowledge and practice to arrive at a composite score may not be entirely reliable in different clime, so it has to be subjected to validation.

## Conclusion

Efforts should be geared towards educating the populace aggressively on facts about COVID-19 including mode of transmission and symptoms, preventive measures and the ills of stigmatization symptoms, and preventive measures across socio demographic groups. Health education in hospitals should be scaled up to fill the information gap in health care processes, as it would help erase wrong beliefs about disease process and its management. Hospital policy should be enunciated to establish telemedicine as part of the framework to patients care why taking into cognizance accessibility in rural and suburban setting.

### What is known about this topic

- It is well established that most patient visiting outpatient urology clinic are middle-aged to elderly, who also have underlying comorbid conditions; they are at risk of severe infections and fatality from coronavirus disease;
- Health literacy is known to affect health seeking behaviour this is further influenced positively by being urban dweller or correlates negatively with rural dwellers.

### What this study adds

- The body of evidence that age is a determinant of health literacy, therefore influenced practice of preventive measure amongst patients visiting outpatient urology clinic positively;
- It was established that there was gap in knowledge on coronavirus disease in terms of health education in our health institution;
- Framework should be developed towards establishing telemedicine with measurable coverage in both urban and suburban settings.
